# Involvement of microRNA Lethal-7a in the Regulation of Embryo Implantation in Mice

**DOI:** 10.1371/journal.pone.0037039

**Published:** 2012-05-18

**Authors:** Wei-Min Liu, Ronald T. K. Pang, Ana W. Y. Cheong, Ernest H. Y. Ng, Kaiqin Lao, Kai-Fai Lee, William S. B. Yeung

**Affiliations:** 1 Department of Obstetrics and Gynaecology, The University of Hong Kong, Pokfulam, Hong Kong, People’s Republic of China; 2 Centre for Reproduction, Development and Growth, The University of Hong Kong, Pokfulam, Hong Kong, People’s Republic of China; 3 Applied Biosystems, Foster City, California, United States of America; Konkuk University, Republic of Korea

## Abstract

MicroRNAs interact with multiple mRNAs resulting in their degradation and/or translational repression. This report used the delayed implantation model to determine the role of miRNAs in blastocysts. Dormant blastocysts in delayed implanting mice were activated by estradiol. Differential expression of 45 out of 238 miRNAs examined was found between the dormant and the activated blastocysts. Five of the nine members of the microRNA lethal-7 (let-7) family were down-regulated after activation. Human blastocysts also had a low expression of let-7 family. Forced-expression of a family member, let-7a in mouse blastocysts decreased the number of implantation sites (let-7a: 1.1±0.4; control: 3.8±0.4) *in vivo*, and reduced the percentages of blastocyst that attached (let-7a: 42.0±8.3%; control: 79.0±5.1%) and spreaded (let-7a: 33.5±2.9%; control: 67.3±3.8%) on fibronectin *in vitro*. Integrin-β3, a known implantation-related molecule, was demonstrated to be a target of let-7a by 3′-untranslated region reporter assay in cervical cancer cells HeLa, and Western blotting in mouse blastocysts. The inhibitory effect of forced-expression of let-7a on blastocyst attachment and outgrowth was partially nullified *in vitro* and *in vivo* by forced-expression of integrin-β3. This study provides the first direct evidence that let-7a is involved in regulating the implantation process partly via modulation of the expression of integrin-β3. (200 words).

## Introduction

Mature microRNAs (miRNAs) are endogenous non-coding RNAs with a size of about ∼21 nt. It has been estimated that miRNAs constitute about 0.5–1% of the genes of different species [1] and that humans may have over 1000 miRNAs (miRBase, April 2011, http://www.mirbase.org/index.shtml). MiRNAs have been found in *Caenorhabditis elegans, Drosophila*, mice and humans, suggesting a conservative role for these RNAs [2]. They bind to target mRNAs inducing their cleavage and/or inhibiting their translation [1], [3], [4]. MiRNAs are involved in diverse biological processes, including morphogenesis and maintenance of tissue, cell growth, differentiation, apoptosis and metabolism [5], [6].

Several observations suggest that miRNAs are involved in early preimplantation embryo development. First, the expression profile of miRNA in preimplantation embryos undergoes dynamic changes during development [7]–[9]. Second, mouse oocytes without a miRNA processing enzyme, dicer possess much reduced amount of miRNAs, disorganized spindle, and their resulting zygotes cannot cleave [7]–[9]. *Dicer*-deficient embryos carrying maternal dicer can only survive to E7.5 [10]. Third, knockdown of Argonaute 2, a catalytic component of the miRNA functioning machinery, RNA-induced silencing complex (RISC), resulted in cleavage arrest of early embryos at the two-cell stage [11]. Fourth, our recent data further confirmed that miR-34c and miR-135a are involved in first cleavage in mouse zygotes [12], [13].

Implantation of blastocysts onto the receptive endometrium is crucial to mammalian reproduction. The process is under endocrine, paracrine, autocrine, and juxtacrine regulation [14]. Recent studies suggest that endometrial miRNAs are involved in the process. MiR-101a and miR-199a* regulate uterine expression of cyclooxygenase-2 around the implantation site [15]. The presence of blastocyst induces the expression of miR-320 and let-7a in the rat uterus during the implantation window [16], [17]. However, there is no study on the role of miRNAs in blastocysts during implantation.

Delayed implantation in mice is an important model for investigation of the implantation process. In this model, dormant blastocysts in the progesterone-primed uterus can be activated within 1 hour after estradiol treatment [18]. The activation is associated with a change in the mRNA expression of blastocysts [19]. We hypothesized that miRNAs were involved in regulating blastocyst implantation. To address this question, we compared the miRNA expression between dormant and activated blastocysts at 3-hour after estradiol treatment in the delayed implantation model. We further demonstrated that one of the differentially expressed miRNAs, let-7a, affects implantation via its action on the expression of integrin in blastocysts.

## Materials and Methods

### Animals and Embryo Collection

The protocol of this study was approved by the Committee on Use of Live Animals in Teaching and Research, the University of Hong Kong (Approval no. 2070-10). ICR female mice aged 6–8 weeks were mated with fertile males. The morning of finding a vaginal plug was designated as Day 1 of pregnancy. To induce delayed implantation, pregnant mice were ovariectomized in the morning (0800–0900 h) of Day 4 and maintained by daily subcutaneous injection of progesterone (2 mg/mouse) from Days 5–7 [18]. Dormant blastocysts were activated by injection of estradiol (25 ng/mouse) into progesterone-primed delayed implanting pregnant mice on Day 7 [19]. Blastocysts were recovered by flushing the uteri with M2 medium at 3–4 hours post-estradiol injection, and were stored at −80°C until use.

Fresh human oocytes and cryopreserved embryos were donated from couples attending the assisted reproduction program at Queen Mary Hospital. The couples gave written consent to the donation of oocytes when no spermatozoa could be isolated from the husband’s testicular biopsies on the day of oocyte retrieval and to the donation of embryos when the couples had a completed family following assisted reproduction treatment. The Institutional Review Board of the University of Hong Kong/Hospital Authority Hong Kong West Cluster approved the protocol (Approval no. UW07-067).

### MiRNA Profiling

Three batches of dormant and activated blastocysts in groups of 5 were collected. The embryos were lysed in 0.5 µl of 2 M guanidine isothiocyanate at room temperature for 5 minutes before the samples were diluted to 5 µl with distilled water for the 238-plex miRNA assay (Applied Biosystems, Foster City, CA). Human oocytes and embryos at different stages of development (2-cell, 4-cell, 8-cell embryos, morulae and blastocysts) were collected and lysed using the same method. Their miRNA expression profiles were determined by the 330-plex miRNA assay (Applied Biosystems). Both assays consisted of three steps: reverse transcription, stem-loop preamplification and quantitative polymerase chain reaction (qPCR) [20]. Reverse transcription was performed in a 10 µl solution containing 1 µl of 10× cDNA Archiving kit buffer, 100 U of MMLV reverse transcriptase, 5 mM of dNTP, 2.6 U of RNase inhibitor, 50 nM of reverse transcription primers for the studied miRNAs, 3.425 mM of MgCl_2_ and 4.5 µl of total RNA. The reaction conditions were: 16°C for 30 minutes, followed by 60 cycles of 20°C for 30 seconds, 42°C for 30 seconds and 50°C for 1 second. The enzyme was eventually inactivated at 85°C for 5 minutes.

Stem-loop preamplification involved amplification of the cDNA in 50 µl of pre-PCR solution containing 25 µl of 2× TaqMan® Universal Master Mix with no AmpErase® UNG, 10 µl of cDNA product, 50 nM of 238-plex forward primer for each miRNA, 5 nM of universal reverse primer, 12.5 U of AmpliTaq Gold, 2 mM of dNTP, 2 mM of MgCl_2_ and 3 µl of ddH_2_O. The PCR condition was 95°C for 10 minutes, 55°C for 2 minutes, followed by 18 cycles of 95°C for 1 second and 65°C for 1 minute.

Quantitative PCR was performed with 50 µl of the pre-amplified sample, which was diluted to 300 µl with water. The qPCR mixture contained 5 µl of 2× Universal Master Mix with no AmpErase®UNG, 0.5 µM of forward primer and 1 µM of TaqMan probe mixture, 1 µM of universal reverse primer, 1 µl of diluted pre-amplified RT-PCR sample, and 0.9 µl of double distilled water. The qPCR condition was 95°C for 10 minutes, followed by 40 cycles at 95°C for 15 seconds, and 60°C for 1 minute. The reactions were performed in an ABI 7500 Real-time PCR System.

### Preparation of Mouse Integrin Beta 3 cRNA

The expression construct of full-length integrin beta 3 (itgb3) was kindly provided by Prof. B.A. Imhof (Centre Medical Unversitaire, Switzerland) [21]. The full-length cDNA of mouse itgb3 was cloned into the BamH1/XhoI site of pcDNA3 which contains a T7 promoter. Plasmid containing itgb3 sequence was linearized. cRNA was synthesized by the mMessage mMachine T7 kit (Ambion, Austin, TX) and purified with the Rneasy Mini Kit (Qiagen) according to the manufacture’s protocol.

### Electroporation and Uterine Embryo Transfer

The oviducts of Day 3 pregnant mice were flushed with Hank’s solution to obtain early 8-cell embryos, which were washed thoroughly and transferred to pre-warmed droplets of KSOM/AA culture medium (Chemicon, Billerica, MA) overlaid with paraffin oil. Precursor of let-7a (pre-let-7a), scramble control (Exiqon, Denmark) or itgb3 RNA, were electroporated into the 8-cell embryos in a flat electrode chamber (1 mm gap between electrodes) (BTX Inc., San Diego, CA) in 20 µl of hepes-buffered saline (150 mM NaCl, 20 mM HEPES) by two sets of 3 electric pulses of 1 ms at 30 V using a 830 Electro Square Porator (BTX Inc.). Following electroporation, the embryos were cultured in fresh KSOM/AA medium. The survival rate of the electroporated embryos was calculated as the percentage of embryos with no sign of cell lysis at 2-hour post-electroporation relative to the total number of electroporated embryos. After 36 hours of culture, 5 blastocysts were transferred into each uterine horn of Day 3 pseudo-pregnant female mice. For assessing the mature let-7a level in embryos upon pre-let-7a electroporation, quantitative PCR was performed on embryos at 24-hour post-electroporation as described [12], [13]. To determine the level of itgb3 protein upon forced-expression, Western blotting was performed on embryos at 24-hour post-electroporation.

### Mouse Embryo Attachment and Outgrowth Assay

Attachment and outgrowth assays were conducted on plastic culture dishes precoated with fibronectin (FN-120) as described [22]. The plastic surface was rinsed three times with fresh culture medium before use. The zona pellucida of blastocysts was removed by acid tyrode. The denuded blastocysts were cultured in microdrops on the fibronectin-coated plate for 72 hours. To determine attachment, the plate was shaken at a rate of one rotation/second on an orbital shaker for 20 seconds. Blastocysts that remained at the same location were designated as attached blastocysts. Outgrowth was identified as the area covered by trophoblast monolayer around the embryo under an inverted microscope with phase contrast optics. The outgrowth area was measured by capturing the image of the outgrowth of 10–15 blastocysts followed by analysis of the images using the Image-Pro software (Media Cybernetics, Inc., MD). Each experiment was repeated three times.

### Luciferase Reporter Assay

A 996 bp oligonucleotide corresponding to a fragment of the 3′-untranslated region (UTR) of mouse itgb3 was synthesized according to the sequence of potential binding regions predicted by two online miRNA target prediction programs, MiRanda (http://www.microrna.org/microrna/) and TargetScan (http://www.targetscan.org/). Specific primers (Forward: 5′-CAGGTTTAACGTGGTAGAAAGTGCTTGACG-3′ and Reverse: 5′-CAGGCGGCCGCGCACGTTGTTCAACAGACTC-3′) for PCR were manufactured by Invitrogen (Carlsbad, CA). The PmeI and NotI restriction enzyme digested and purified amplicons were cloned downstream of the Renilla luciferase gene within the PmeI-NotI treated psiCHECK-2 vector (Promega, Madison, WI) to generate the wild type reporter construct. The positive clone was two-nucleotide-point mutated at the let-7a complementary site using the QuikChange II Site Directed Mutagenesis Kit (Stratagene, La Jolla, CA) according to the manufacturer’s protocol. The mutant sequence was 5′…TGTGTATCgAgCTCTTCGGTGTCTT…3′. Relative luciferase activities were determined by normalizing the renilla luciferase activity against the firefly luciferase activity.

### Western Blot Analysis

Western blotting was performed as previously described [23]. In brief, 40 blastocysts were pooled and rinsed twice in PBS, resuspended in 10 µl of Laemmli Buffer [24] and boiled for 10 minutes. The samples were run in 10% SDS-PAGE gels. To increase the sensitivity of the assay, the samples in each lane was concentrated by reducing the width of the sample wells to 2 mm with a home-made sample comb. Protein bands in the gel were transferred onto nitrocellulose membranes, which were incubated with the primary antibodies in Tris-buffered saline containing 5% nonfat milk and 0.5% Tween 20 (TBST) overnight at 4°C, followed by incubation with appropriate horseradish peroxidase conjugated secondary antibody (1∶1000) in TBST for 1 hour. The bands were detected using an enhanced chemiluminescence kit (Santa Cruz Biotechnology, Santa Cruz, CA).

### Data Analysis

Statistical analysis of the 238-plex miRNA data was performed with the GeneSpring™ software version 7.3 (Sigenetics Inc., San Carlos, CA). One-way ANOVA non-parametric t-test for paired comparison was applied. A difference with p-value of less than 0.05 was considered as significant. Values for each miRNA were median-centered before clustering. For analysis of the possible function of the miRNAs differentially expressed between the dormant and activated blastocysts, their potential target genes were searched using TargetScan. The predicted target gene list from the up- and the down-regulated miRNAs were grouped together and subjected to functional analysis by PANTHER [25] using *Mus musculus* gene list as background.

## Results

### Activation of Dormant Blastocysts is Associated with Changes in microRNAs Expression

**Figure 1:**
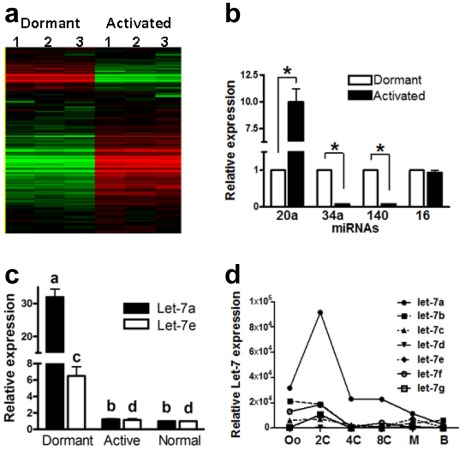
MicroRNA profiling of dormant and activated mouse blastocysts. (a) Supervised hierarchical clustering of the expression of 238 miRNAs in mouse dormant and activated blastocysts. Heat map shows normalized, log-transformed relative intensities of the studied miRNA. Red, green and black color represent low, high and mean expression levels of miRNAs, respectively. Genes with similar expression patterns are grouped together. (b) Validation of expression of miR-34a, -20a, -140, and -16 by qPCR without pre-amplification. The expression patterns of these miRNAs in dormant and activated blastocyst were similar to those determined by miRNA profiling. All values are calculated against the C_T_ values of dormant blastocysts and are presented as relative fold-change. (c) Relative expression of let-7a and let-7e in dormant, activated and normal Day 4 blastocysts as determined by qPCR without pre-amplification (^a–b, c–d^ denote P<0.05 between corresponding groups). (d) The expression of let-7 in human embryos.

The 238-multiplex kit specific for mouse miRNAs was used to determine the miRNAs profiles in dormant and activated blastocysts. To reduce variation, groups of 5 blastocysts were used for each datum, and the experiment was repeated thrice. The relative levels of miRNA expression in the dormant and the activated blastosysts were compared by the average difference values. Supervised hierarchical clustering analysis using the Genespring software is shown in **Fig. 1a**. One way ANOVA analysis revealed that 45 miRNAs (18.9%) were differentially expressed between the dormant and the activated blastocysts (P<0.05). Of these miRNAs, 38 were up-regulated and 7 were down-regulated in the dormant blastocysts when compared with the activated blastocysts (Table 1). Several miRNA clusters were observed among the differentially expressed miRNAs. These include the miR-17∼92 (miR-17-5p, -18, -19b, -20), 15a/16 (miR-15a, -15b), and 290–295 (miR-290, -294, -295) clusters. Several members of the let-7 family (let-7a, -7d, -7e, -7f, -7g) were also found in the list.

**Table 1:** MicroRNAs differentially expressed between dormant and activated blastocysts (p<0.05).

Up-regulated miRNA	fold change (activated/dormant)	Down-regulatedmiRNA	fold change (activated/dormant)
miR-376b	505610	miR-410	0.52
miR-20	16.07	miR-200b	0.46
miR-133a	9.25	miR-101b	0.43
miR-199a	5.20	miR-350	0.44
miR-483	2.78	miR-339	0.40
miR-181a	2.54	miR-141	0.29
miR-324-3p	2.22	miR-15b	0.26
		let-7g	0.25
		miR-26b	0.25
		miR-292-5p	0.22
		miR-18	0.20
		miR-19b	0.18
		miR-155	0.18
		miR-196b	0.17
		miR-295	0.17
		miR-424	0.17
		let-7d	0.16
		miR-203	0.16
		miR-10b	0.14
		miR-345	0.14
		miR-26a	0.12
		miR-328	0.12
		miR-341	0.12
		miR-297	0.11
		miR-15a	0.10
		miR-127	0.09
		miR-294	0.08
		miR-429	0.08
		let-7e	0.07
		miR-24	0.07
		miR-324-5p	0.05
		let-7a	0.03
		let-7f	0.03
		miR-34a	0.03
		miR-140	0.02
		miR-17-5p	0.01
		miR-290	0.01
		miR-149	0.0003

To confirm the data obtained from the miRNA profiling experiment, total RNAs isolated from 5 pools of dormant and activated blastocysts were subjected to direct qPCR analysis without pre-PCR amplification for 4 miRNAs. The results (**Fig. 1b**) confirmed that the expression patterns of miR-20a, miR-34a, miR-140 and miR-16 were consistent with those obtained after pre-PCR amplification. Apart from miR-16, significant differences in the levels of these miRNAs were found between the dormant and the activated blastocysts.

The potential target genes of the differentially expressed miRNAs as predicted by TargetScan were subjected to biological processes and pathway analysis by PANTHER [26]. The top 10 biological processes and signaling pathways are shown in Tables 2 and 3, respectively. The biological processes predicted from the target genes of both up- and down-regulated miRNAs (P<10^−26^) were identical and included metabolic processes, cellular and developmental process, cell communication, cell cycle, signal transduction and intracellular signaling cascade, consistent with the expected difference in biological activities between the dormant and the activated blastocysts. In the pathway analysis, Wingless (Wnt) signaling, transforming growth factor-β (TGFβ) signaling, epidermal growth factor (EGF) receptor signaling, Rat Sarcoma (Ras), integrin signaling and platelet-derived growth factor (PDGF) signaling, phosphoinositide 3-kinase (PI3-K) and angiogenesis pathways were predicted from both the up- and the down-regulated miRNAs (P<10^−7^).

**Table 2:** Biological processes of the differentially-regulated miRNAs.

Biological Process	Genes (REF)	Targets of miRNAs			
	#	#	expected	+/−	P value
**Up-regulated miRNAs**					
Primary metabolic process	9122	878	572.72	+	1.72E-53
Nucleobase, nucleoside, nucleotide and nucleic acid metabolic process	4156	499	260.93	+	1.01E-48
Metabolic process	9603	891	602.92	+	2.48E-47
Cellular process	7133	717	447.84	+	4.55E-46
Developmental process	3296	405	206.94	+	2.00E-40
Intracellular signaling cascade	1720	237	107.99	+	2.41E-29
Signal transduction	4858	488	305.00	+	6.88E-28
System development	2222	277	139.51	+	1.16E-27
Cell communication	5033	500	315.99	+	1.33E-27
Cell cycle	2018	255	126.70	+	3.38E-26
**Down-regulated miRNAs**					
Cellular process	7133	1658	1031.06	+	9.47E-106
Primary metabolic process	9122	1967	1318.57	+	3.78E-103
Metabolic process	9603	2000	1388.10	+	5.14E-91
Nucleobase, nucleoside, nucleotide and nucleic acid metabolic process	4156	1034	600.74	+	2.25E-71
Developmental process	3296	857	476.43	+	2.24E-65
Cell communication	5033	1152	727.51	+	2.84E-61
Signal transduction	4858	1101	702.22	+	8.56E-56
Intracellular signaling cascade	1720	509	248.62	+	9.15E-52
System development	2222	606	321.19	+	8.21E-51
Cell cycle	2018	563	291.70	+	7.75E-50

**Table 3:** Pathways of differentially-regulated miRNAs.

Pathways	Genes (REF)	Targets of miRNAs			
	#	#	expected	+/−	P value
**Up-regulated miRNAs**					
TGF-beta signaling pathway	143	40	8.98	+	1.98E-14
Angiogenesis	193	41	12.12	+	4.67E-11
EGF receptor signaling pathway	138	34	8.66	+	4.88E-11
PDGF signaling pathway	162	37	10.17	+	5.71E-11
PI3 kinase pathway	110	29	6.91	+	2.76E-10
Integrin signalling pathway	185	38	11.62	+	5.87E-10
Interleukin signaling pathway	160	33	10.05	+	7.00E-09
FGF signaling pathway	125	28	7.85	+	1.75E-08
Insulin/IGF pathway-protein kinase B signaling cascade	83	22	5.21	+	3.41E-08
Ras Pathway	80	21	5.02	+	8.10E-08
Insulin/IGF pathway-mitogen activated protein kinase kinase/MAP kinase cascade	36	14	2.26	+	1.23E-07
Wnt signaling pathway	348	50	21.85	+	1.36E-07
**Down-regulated miRNAs**					
Integrin signalling pathway	185	87	26.74	+	1.50E-20
EGF receptor signaling pathway	138	72	19.95	+	1.33E-19
FGF signaling pathway	125	67	18.07	+	6.33E-19
Angiogenesis	193	83	27.90	+	1.86E-17
PDGF signaling pathway	162	68	23.42	+	4.25E-14
Wnt signaling pathway	348	111	50.30	+	6.70E-14
Ras Pathway	80	44	11.56	+	2.50E-13
TGF-beta signaling pathway	143	58	20.67	+	1.12E-11
PI3 kinase pathway	110	47	15.90	+	1.84E-10
p53 pathway	127	51	18.36	+	2.62E-10

### The Expression of Let-7a is Low in Blastocyst before Implantation

Nine members of the let-7 family were analyzed in the profiling experiment. Five of them (let-7a, -7d, -7e, -7f and -7g) were down-regulated by more than 2-fold in the activated blastocysts (Table 4). The expression of two members (let-7c and -7i) were below the detection limit while that of the rest (let-7d* and -7b) did not change after activation. To confirm the observation, the levels of let-7a and -7e in normal, dormant and activated blastocysts were compared by direct qPCR. The results showed that their expression levels were significantly higher in the dormant blastocysts than the other 2 types of blastocysts (**Fig. 1c**).

**Table 4:** Expression of let-7 family members (in Ct values) in dormant and activated blastocysts.

MiRNA	Dormant (Dor)				Activated (Act)				fold change Act/Dor
	1	2	3	Mean	1	2	3	Mean	
mmu-let-7b	18.5	18.0	18.4	18.3	18.6	18.8	18.9	18.8	0.72
mmu-let-7c	BD	BD	BD	−	BD	BD	BD	−	−
mmu-let-7d	26.7	26.3	26.5	26.5	BD	BD	BD	−	∞
mmu-let-7e	20.7	20.8	20.9	20.8	24.7	24.5	24.7	24.6	0.07
mmu-let-7i	BD	BD	BD	−	BD	BD	BD	−	−
mmu-let-7g	21.6	21.9	21.6	21.7	23.6	23.7	23.9	23.7	0.25
mmu-let-7a	21.2	21.4	21.0	21.2	26.2	26.6	26.5	26.4	0.03
mmu-let-7d*	18.9	18.5	18.2	18.5	19.0	18.9	18.6	18.8	0.80
mmu-let-7f	19.0	19.5	19.2	19.2	24.6	23.9	24.0	24.2	0.03

BD =  below detection limit.

To determine whether a low level of let-7 also occurred in human blastocysts, donated human embryos cryopreserved at the 2–4-cell stage were thawed and cultured to blastocysts. Individual donated fresh human oocytes and cultured human embryos at 2-cell, 4-cell, 8-cell, morula and blastocyst stages were subjected to the 330-multiplex miRNA assay (n = 3 for each). Similar to the report in mouse [9], a continuous decrease of expression levels of let-7 family members was found during preimplantation development of human embryos (**Fig. 1d**).

### Let-7a Inhibits Embryo Implantation in vivo

**Figure 2:**
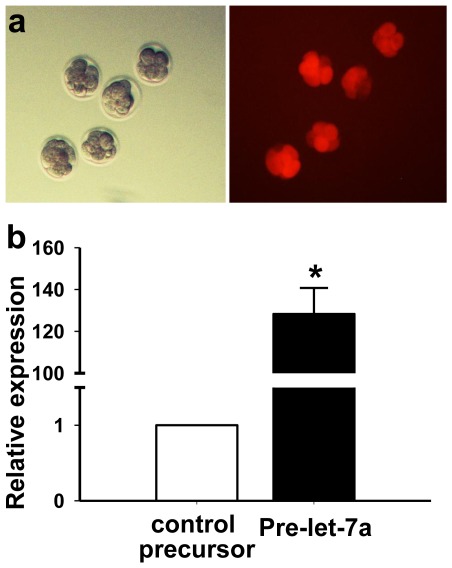
Electroporation of RNA at the 8-cell embryo stage. (a) Electroporation of Cy3-labeled scramble RNA. Fluorescence was detected in all the electroporated embryos. (b) Relative level of mature let-7a in embryos after electroporation of pre-let-7a at the 8-cell embryo stage. Each experiment contained 5 embryos and the experiment was repeated at least 3 times. (*P<0.05, One-way ANOVA).

In view of the high level of let-7 in dormant embryos, we postulated that up-regulation of let-7 suppress blastocyst implantation. Here, we studied the let-7 function by forced expression of precursor of let-7 in embryos using electroporation as described for studying the roles of specific genes in mouse preimplantation embryo development [27], [28]. The optimal conditions for electroporation of 8-cell embryos in microslide chamber was found to be two series of three 1 ms pulses of 30 V. Using Cy3-labeled scramble RNA as a visual indicator of successful electroporation, fluorescence was detected in all the electroporated embryos (**Fig. 2a**). The survival rate of the electroporated embryos was more than 95% under such conditions. Eighty-two percent of the electroporated embryos developed to blastocysts after 30 hours of culture. The blastulation rates were similar between blastocysts electroporated with pre-let-7a and those with control RNA.

**Figure 3:**
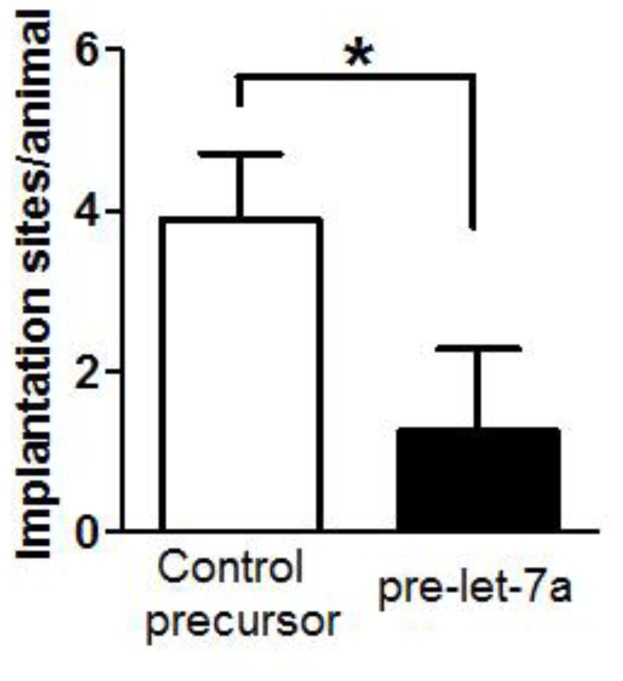
Precursor of let-7 inhibits embryo implantation *in vivo*. Eight-cell embryos were electroporated with precursor of let-7a or control RNA, and transferred into opposite uterine horns of mice on Day 3 of pseudopregnancy (5 embryos for each horn). Let-7a reduced the number of embryos implanted in the uterus. Statistical significant difference (P<0.05, Chi square) in the number of implantation sites was found on Day-8 of pregnancy (n = 18).

To investigate the role of let-7a in implantation of blastocysts, 8-cell embryos were electroporated with pre-let-7a or control precursor. After 30 hours of culture, the blastocysts derived from embryos electroporated with pre-let-7a expressed 110 times more mature let-7a than those with control RNA (**Fig. 2b**). The two groups of blastocysts were transferred into opposite uterine horns (5 embryos per horn) of Day 3 pseudopregnant mice. The numbers of implantation sites were determined on Day 8 of pregnancy. We found that forced-expression of let-7a in embryos resulted in a significant reduction in the number of implantation sites (1.1±0.4) when compared with those electroporated with control precursor (3.8±0.4) (**Fig. 3**).

### Let-7a Inhibits Embryo Attachment and Outgrowth in vitro

**Figure 4:**
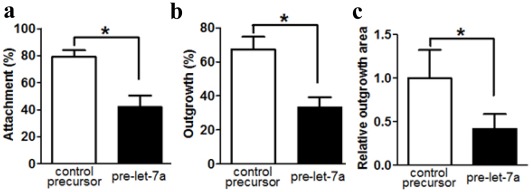
The effects of let-7a on the embryo attachment, percentage outgrowth and outgrowth area *in vitro*. Eight-cell embryos were electroporated with precursor of let-7a or control RNA, and cultured on fibronectin coated dishes. (a) Let-7a inhibited blastocyst attachment on fibronectin after 36-hour of culture. (b) let-7a reduced the number of attached blastocysts showing trophoblast outgrowth rate after 48-hour of culture. (c) let-7a decreased the trophoblast outgrowth area after 72-hour of culture. Each experiment consisted of 25–30 embryos and the experiment was repeated at least 5 times (*P<0.05, One-way ANOVA).

In order to confirm the action of let-7a on the implantation process, 8-cell embryos were electroporated with pre-let-7a or control precursor, and the ability of the resulting blastocysts attaching onto fibronectin was determined. At 36-hour post-electroporation, the percentage of attached blastocysts with forced-expression of let-7a (42.0±8.3%) was significantly lower than that of blastocysts with control precursor (79.0±5.1%) (**Fig. 4a**). The percentage of the former group of blastocysts with trophoblast outgrowth (33.5±2.9%) was also lower than that of the latter group (67.3±3.8%) at 48-hour post-electroporation (**Fig. 4b** **)**, at which most of the blastocysts electroporated with pre-let-7a exhibited only early signs of spreading. The trophoblast migratory activity was quantified by the area of trophoblast outgrowth at 72-hour post-electroporation. The results showed that the outgrowth area was also significantly decreased with let-7a forced-expression (pre-let-7a 54885±9880 µm^2^ vs control 26753±4976 µm^2^; **Fig. 4c**).

### Let-7a Directly Targets Mouse Integrin-β3 Subunit

**Figure 5:**
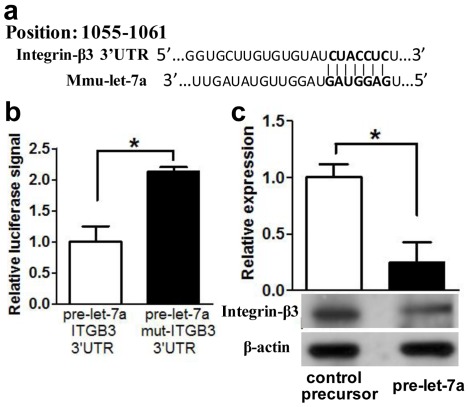
Let-7a interacts with the 3′-untranslated region (UTR) of integrin β3. (a) The potential let-7a binding site in mouse integrin-β3 (Itgb3). (b) Luciferase activities of the wild type or mutated integrin-β3 3′UTR reporter constructs co-electroporated with the let-7a precursor in HeLa cells. Dual luciferase assays were performed in cell lysates at 48-hour post-electroporation. (c) Western blot analysis of integrin-β3 protein from blastocysts electroporated with pre-let7a or control RNA at the 8-cell embryo stage. Each lane contained extract from 40 blastocysts. Semi-quantitative analysis of integrin-β3 expression levels in the embryos. There was a significant decrease in integrin-β3 protein expression in embryos eclectroporated with pre-let-7a relative to the control. The data represent the mean and standard errors (n = 3). *denotes P<0.05.

TargetScan and MiRanda predicted integrin-β3 as a potential target gene of let-7a (**Fig. 5a**). To characterize the relationship between let-7a and integrin-β3 expression, wild type or mutated reporter construct was co-electroporated with the let-7a precursor into HeLa cells for dual luciferase assay. Cells electroporated with let-7a precursor significantly suppressed the wild type reporter activity by 50% (**Fig. 5b**). This effect was abrogated when the mutated reporter construct was used. Electroporation of the cells with the empty reporter vector or co-electroporating them with the scramble controls did not affect the luciferase activities. The results demonstrated that let-7a directly interacted with the 3′UTR of mouse integrin-β3.

To confirm that let-7a also regulated integrin-β3 expression in blastocysts, the expression of integrin-β3 protein in blastocysts at 36-hour post-electroporation of either let-7a precursor or control RNA was examined. Western blot analysis showed that the let-7a precursor decreased the expression of integrin-β3 protein in blastocysts (**Fig. 5c**).

### Forced-expression of Integrin-β3 Partially Rescues the Inhibitory Effects of Let-7a

**Figure 6:**
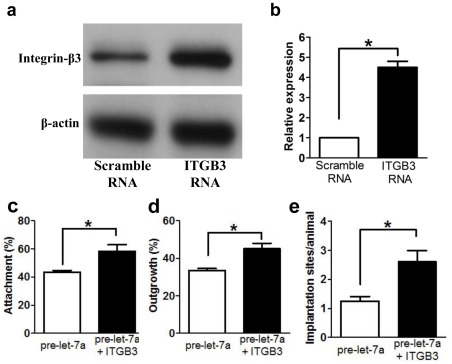
Forced-expression of integrin-β3 partially nullifies the inhibitory action of let-7a on implantation. Eight-cell embryos were electroporated with let-7a precursor either with or without integrin β3 (Itgb3) RNA. (a) Expression of Itgb3 protein after electroporation. Each lane contained protein lysate from 40 blastocysts. The experiment was repeated 3 times. (b) Semi-quantitative comparison of the Itgb3 protein expression. (c) Attachment and (d) spreading of the resulting blastocysts *in vitro*. Forced-expression of integrin β3 partially reduced the inhibitory effects of let-7a on embryo attachment and outgrowth. Each datum point consisted of 25–30 embryos. The data represent the mean and standard errors (n = 3). (e) Implantation of the electroporated blastocyst after transfer to the uterine horn on day 3 of pseudopregnancy. The number of implanted embryos was counted on Day 8 of pregnancy. Five blastocysts were transferred to each horn (n = 14). Forced-expression of integrin β3 partially reduced the inhibitory effects of let-7a on implantation *in vivo*. *denotes P<0.05 (One way ANOVA).

To determine whether integrin-β3 mediated the action of let-7a on embryo implantation, ectopic expression of integrin-β3 was used to nullify the inhibitory activity of let-7a precursor. The level of integrin-β3 protein significantly increased in embryos electroporating integrin-β3 RNA after 24 hours of culture in vitro (**Fig. 6a,b**). Forced-expression of integrin-β3 did not affect the percentages of embryo that attached (integrin β3 82.5±7.8% vs control 81.2±11.1%) and spread (integrin β3 69.3±8.1% vs control 67.1±6.5%) on fibronectin. Electroporation of pre-let-7a alone inhibited the ability of the embryo for attachment and spreading, while co-electroporation with integrin-β3 RNA partially reduced these suppressive actions of pre-let-7a (**Fig. 6c and d**). Forced-expression of integrin-β3 also reduced the inhibitory action of pre-let-7a on implantation *in vivo* (**Fig. 6e**).

## Discussion

We hypothesize that miRNAs regulate gene and protein expression during activation of dormant blastocysts. Comparison of the miRNA profile in dormant and activated blastosysts showed differential expression of 45 miRNAs. Vast majority (38/45) of them were down-regulated after activation. As miRNAs suppress translation, the presence of more up-regulated miRNAs in the dormant blastocysts was consistent with the inactive nature of these embryos. Bioinformatic analysis of the differentially expressed miRNAs showed that their potential targets are involved in biological processes known to be implicated in dormancy and its subsequent activation.

Dormancy is associated with reduction in DNA synthesis, cell cycling and metabolism [29], [30]. In the delayed implantation mouse model, estrogen-induced activation stimulates mitosis and increases cell number of the dormant blastocysts *in vivo* [31], consistent with the predicted involvement of the potential target genes of the differentially expressed miRNAs in cell cycle, nucleic acid metabolism and metabolic processes. Indeed some of the identified miRNAs have been shown to be related to these processes in other biological systems. Interestingly, these miRNAs are all down-regulated in the activated blastocysts relative to the dormant blastocysts. The expression of miR-34a decreased by 128-fold after activation. Its ectopic expression induces cell cycle arrest in both primary and tumor-derived cell lines by down-regulation a panel of genes that promote cell cycle progression [32]. Two members of the miR-15a/16 cluster, miR-15a and miR-15b, were also down-regulated in the activated blastocysts. MiR-34a acts synergistically with these miRNAs to induce G_1_–G_0_ arrest in lung cancer [33]. Forced-expression of miR-26a, another down-regulated miRNA in activated blastocysts, also induces cell cycle arrest in nasopharyngeal carcinoma [34]. The dormant blastocysts have reduced rate of glucose oxidation caused by allosteric inhibition of the rate-controlling enzyme for glycolysis, phosphofructokinase [35]. Incidentally, the enzyme is a predicted target (TargetScan) of miR-295, of which the expression is high in the dormant blastocysts. The observation is also consistent with the detection of a significant increase in the expression of phosphofructokinase mRNA in the activated blastocysts [19].

MiR-290-295 cluster consists of eight members. Half of them (miR-290, -292-5p, -294, -295) were down-regulated in the delayed implanting blastocysts after estrogen administration. The cluster is expressed during early embryogenesis and is mouse embryonic stem cells specific [36]. The cluster inhibits differentiation of mouse embryonic stem cells, and is down-regulated during differentiation [37], [38]. The down-regulation of this cluster after activation may be required for preparation of the activated blastocysts to implant and to differentiate.

Three members of the miR-17-92 clusters (miR-17-5p, -19b, -18) were down-regulated after activation. In cancer, up-regulation of miR-17-92 inhibits TGFβ signaling [39], [40]. Another down-regulated miRNA, miR-140, also suppresses the TGFβ pathway via repression of Smad3 in the murine multipotential mesenchymal cells [41]. In mouse blastocysts, TGFβ binds to the trophectoderm [42] and regulates trophoblast outgrowth [43]. It is interesting to note that TGFβ immunoreactivities are absent in the delayed implanting blastocysts but reappeared after activation [42], [44], [45]. The contribution of the above miRNAs to this change in the TGFβ signaling needs to be confirmed.

Apart from TGFβ signaling, three other pathways, EGF receptor signaling, Wnt signaling and PI3 kinase pathway, predicted by bioinformatic tools were known to be involved during activation of dormant blastocysts. Activation of dormant blastocysts is associated with increase of EGF receptor signaling [46]. The trophectoderm function of activated mouse blastocysts requires a coordinated activation of the canonical Wnt-β-catenin signaling and an inhibition of the non-canonical Wnt-RhoA signaling [47]. A global mRNA profiling study shows that genes involved in PI3-K signaling including Pik3c2a are increased in activated blastocysts [19]. The regulation of these pathways in the delayed implanting blastocyst by the differentially expressed miRNAs remains to be determined.

MiRNAs inhibit the translation of their targets. Hamatani and coworkers [19] compared the mRNA expression profiles of dormant and activated mouse blastocysts. Among the 135 differentially expressed mRNAs identified in the study, only 17 (12.6%) of them are common with the predicted targets of the differentially expressed miRNAs in the present study. The low percentage of common mRNAs in the two studies probably reflects the difference in sampling time. The samples were taken at 3–4 hours post-estrogen administration in the present study whereas samples at 12–14 hours post-estrogen administration were used in Hamatani and coworkers’ study.

The let-7 family consists of 11 members, which are conserved in invertebrates and vertebrates, including humans [48], [49]. The expression of 9 family members was studied. Five of them (let-7a, -7d, -7e, -7f and -7g) were down-regulated by more than 2-fold when dormant blastocysts were activated. Let-7 family is widely demonstrated as a tumor suppressor. Its expression is down-regulated in many cancers when compared to normal tissue [50]–[52]. Let-7 controls cellular proliferation by negatively regulating RAS and cell cycle-related genes such as cyclin D2, CDK6 and CDC25A [53]. Down-regulation of let-7 in the activated blastocysts would enable up-regulation of let-7-response genes, many of which are oncogenes or cell cycle checkpoint genes, leading to cell cycle progression, DNA synthesis and cell division. Increases in cell number and uridine incorporation in dormant blastocysts are characteristics of activation in utero by estrogen [19].

The expression of let-7 is dynamically regulated during oogenesis and early embryonic development [7]. There is a continuous decrease in its expression from the oocyte to the 8-cell embryo stage [9]. The present study showed that the expression level of let-7 family in human blastocysts is also low, consistent with a similar role of the miRNA in preimplantation embryo development. The high level of let-7 during dormancy relative to the normal blastocyst and the down-regulation of let-7 in the activated blastocysts suggest that a low level of let-7 is beneficial for implantation. Here, we provide the first *in vitro* and *in vivo* evidence that let-7a affects embryo implantation, at least partly through regulation of expression of integrin-β3. The evidence include (a) Let-7a bound to the 3′UTR region of the integrin-β3; (b) forced-expression of let-7a reduced the expression of integrin-β3; and (c) forced-expression of integrin-β3 partially rescued the suppressive effect of let-7 on blastocyst implantation, attachment and outgrowth.

Integrins are a family of heterodimeric (α/β) transmembrane molecules primarily responsible for interaction between the mouse trophoblasts and the extracellular matrix molecules of the endometrium [54]. Apart from cell adhesion, integrin binding transduces intracellular signaling involved in cell migration, differentiation and survival [55]. Blastocyst adhesion to fibronectin requires integrin-mediated activation of phosphoinositide-specific phospholipase C leading to initiation of phosphoinositide signaling and intracellular calcium mobilization [56].

There is only one previous report on integrin expression in dormant blastocysts showing reduced α4β1 expression relative to that of the activated blastocysts [57]. Here we provide evidence that integrin-β3 mediates the action of let-7a on implantation of activated blastocysts. The low level of let-7a in the normal blastocysts and the activated blastocysts would favor the expression of integrin-β3 subunit. It is interesting to note that integrin α_v_ subunit is one of the target genes of the miR-295 cluster [58], which is also down-regulated in the activated blastocysts. These observations suggest that integrin α_v_β3 would be up-regulated in the activated blastocysts, consistent with the report that α_v_β3 is the only integrin among a repertoire of integrins expressed in mouse embryos detected on the apical surface of the trophectoderm of late blastocysts and the proposal that integrin α_v_β3 mediates primary trophoblast adhesion and migration [59].

Differentiation of the trophectoderm cells into trophoblast cells is associated with translocation of the integrins α5β1 [54] and αIIbβ3 [60], [61], and increased binding ability to fibronectin [54]. The fibronectin-binding activity of blastocyst depends on integrins α5β1 and α_v_β3 [62]. The importance of integrins in implantation is shown by significant reduction of implantation after injection of echistatin, which inactivates α_v_β3, α5β1 and αIIbβ3, into the uterine horns [63], [64]. It is likely that these integrins have compensatory function in implantation as mice lacking integrin-β3 are fertile and their embryos are compatible with implantation [65].

Apart from the possible compensatory function of integrins, the inability of forced-expression of integrin-β3 in completely nullifying the inhibitory action of let-7 precursor on blastocyst outgrowth could be due to the involvement of other pathway(s) mediating the action of of let-7 on embryo implantation. Each miRNA can regulate the expression of a large number of genes. Other than integrin-β3, let-7 is known to regulate RAS [66], HMGA2 [67] and Dicer [68]. Among the potential pathways predicted from miRNAs differentially expressed between dormant and activated blastocysts, the Wnt pathway may be relevant to the observed action of let-7. Two miRNAs target gene prediction softwares, TargetScan and PicTar show that Kremen1 and Wnt1 are target genes of let-7. Wnt1 is predominantly expressed in the inner cell mass of mouse blastocyst [69], while Kremen1 is detected primarily in the trophectoderm of dormant blastocysts and is translocated into the nuclei of trophodermal cells in activated blastocysts [47]. The contribution of the Wnt pathway to the action of let-7 is being investigated.

Let-7 family members have also been implicated in the early pregnancy. Several of the let-7 members are upregulated in the delayed implantating mouse uterus after activation [70] and in the implantation site relative to inter-implantation site [71]. Both the luminal epithelium and the stroma of endometrium express let-7 members with unknown function [71], [72]. Integrin-β3 is expressed in the uterine luminal epithelium of mice [73] and rats [74]. Interestingly, the expression of integrin-β3 in the latter [74] but not the former [73] is under steroid control. Whether let-7 modulates the expression of integrin-β3 in the uterine luminal epithelium remains to be determined.

In conclusion, this study reported the differential expression of miRNAs between dormant and activated blastocysts. The results showed that let-7 is involved in the regulation of blastocyst activation. The knowledge gained may be applied to humans as a continuous down-regulation of let-7 is also observed in the human preimplantation embryos. The present investigation provides information on a novel mechanism in the initiation of embryo implantation.
